# Neoadjuvant irinotecan, cisplatin, and concurrent radiation therapy with celecoxib for patients with locally advanced esophageal cancer

**DOI:** 10.1186/s12885-016-2485-9

**Published:** 2016-07-13

**Authors:** James M. Cleary, Harvey J. Mamon, Jackie Szymonifka, Raphael Bueno, Noah Choi, Dean M. Donahue, Panos M. Fidias, Henning A. Gaissert, Michael T. Jaklitsch, Matthew H. Kulke, Thomas P. Lynch, Steven J. Mentzer, Jeffrey A. Meyerhardt, Richard S. Swanson, John Wain, Charles S. Fuchs, Peter C. Enzinger

**Affiliations:** Center for Esophageal and Gastric Cancer, Dana-Farber Brigham and Women’s Cancer Center and Gastrointestinal Cancer Center, Department of Medical Oncology, Dana-Farber Cancer Institute and Harvard Medical School, 450 Brookline Ave, Boston, MA 02215 USA; Massachusetts General Hospital Cancer Center, Harvard Medical School, Boston, MA USA; University of Arizona Cancer Center, St. Joseph’s Hospital and Medical Center, Phoenix, AZ USA

**Keywords:** Esophageal cancer, Neoadjuvant therapy, Chemoradiation, Cyclooxygenase 2 inhibition

## Abstract

**Background:**

Patients with locally advanced esophageal cancer who are treated with trimodality therapy have a high recurrence rate. Preclinical evidence suggests that inhibition of cyclooxygenase 2 (COX2) increases the effectiveness of chemoradiation, and observational studies in humans suggest that COX-2 inhibition may reduce esophageal cancer risk. This trial tested the safety and efficacy of combining a COX2 inhibitor, celecoxib, with neoadjuvant irinotecan/cisplatin chemoradiation.

**Methods:**

This single arm phase 2 trial combined irinotecan, cisplatin, and celecoxib with concurrent radiation therapy. Patients with stage IIA-IVA esophageal cancer received weekly cisplatin 30 mg/m^2^ plus irinotecan 65 mg/m^2^ on weeks 1, 2, 4, and 5 concurrently with 5040 cGy of radiation therapy. Celecoxib 400 mg was taken orally twice daily during chemoradiation, up to 1 week before surgery, and for 6 months following surgery.

**Results:**

Forty patients were enrolled with stage IIa (30 %), stage IIb (20 %), stage III (22.5 %), and stage IVA (27.5 %) esophageal or gastroesophageal junction cancer (AJCC, 5th Edition). During chemoradiation, grade 3–4 treatment-related toxicity included dysphagia (20 %), anorexia (17.5 %), dehydration (17.5 %), nausea (15 %), neutropenia (12.5 %), diarrhea (10 %), fatigue (7.5 %), and febrile neutropenia (7.5 %). The pathological complete response rate was 32.5 %. The median progression free survival was 15.7 months and the median overall survival was 34.7 months. 15 % (*n* = 6) of patients treated on this study developed brain metastases.

**Conclusions:**

The addition of celecoxib to neoadjuvant cisplatin-irinotecan chemoradiation was tolerable; however, overall survival appeared comparable to prior studies using neoadjuvant cisplatin-irinotecan chemoradiation alone. Further studies adding celecoxib to neoadjuvant chemoradiation in esophageal cancer are not warranted.

**Trial registration:**

Clinicaltrials.gov: NCT00137852, registered August 29, 2005.

## Background

Locally advanced esophageal cancer is an aggressive malignancy with a high recurrence rate [1]. Meta-analyses of neoadjuvant chemoradiation trials suggest that there is a survival benefit for patients treated with neoadjuvant chemoradiation and surgery compared to patients undergoing surgery alone [2, 3]. Moreover, multiple studies have reported that pathological complete response after neoadjuvant chemoradiation predicts increased survival [4–7].

Two prior randomized clinical trials testing neoadjuvant radiotherapy with cisplatin and 5-FU followed by surgery demonstrated a survival benefit compared to patients treated with surgery alone [8, 9]. Recently, the CROSS trial demonstrated a significant survival benefit for neoadjuvant radiotherapy with carboplatin and paclitaxel followed by surgery when compared to surgery alone, rendering this regimen as a widely used standard of care. Patients treated on the CROSS trial had a median overall survival of 49 months and a pathological complete response rate of 29 % [10].

Cisplatin/irinotecan is an active regimen in advanced esophageal cancer [11, 12]. Neoadjuvant chemoradiation with cisplatin/irinotecan has also been evaluated in two phase 2 studies. Both trials reported a 16 % pathological complete response rate; median survival was 31.7 and 36 months, respectively [13, 14]. The major toxicities of cisplatin/irinotecan and radiation therapy were myelosuppression, esophagitis, and diarrhea. While there have been multiple trials testing neoadjuvant chemoradiotherapy prior to surgery compared to surgery alone in locally advanced esophageal cancer, there are no trials comparing chemotherapy combinations to use with radiation and thus there is no one standard backbone regimen to incorporate in trials with targeted therapies.

Several lines of study suggest that non-steroidal anti-inflammatory (NSAID) medications modify the natural history of selected gastrointestinal malignancies and that inhibition of cyclooxygenase 2 (COX2) plays an important role in this effect. In colorectal cancer, aspirin and NSAIDs appear to increase survival and decrease the risk of cancer development and recurrence [15–19]. Increased COX2 expression in esophageal cancer has also been associated with decreased survival and is thought to play a role in promoting the progression from Barrett’s esophagus to esophageal adenocarcinoma [20–24]. Several preclinical studies have shown that the selective COX2 inhibitor celecoxib works synergistically with radiation to increase cancer cell death and high expression of COX2 correlates with decreased response to radiation [25–29].

Based on these data, we conducted a phase II trial of celecoxib in conjunction with neoadjuvant radiation therapy and concurrent cisplatin plus irinotecan in patients with resectable locally advanced esophageal cancer.

## Methods

### Trial design

This phase 2 clinical trial (NCT00137852) was a single arm study evaluating the efficacy and safety of perioperative celecoxib and neoadjuvant chemoradiation with weekly cisplatin plus irinotecan followed by surgery in locally advanced esophageal and gastroesophageal junction cancer patients.

### Study population

This trial was open to patients with stage IIA, IIB, III, IVA esophageal or gastroesophageal junction cancer by 5th American Joint Committee on Cancer (AJCC) [30]. Both adenocarcinoma and squamous cell carcinoma histologies were permissible. All patients underwent staging workup with a CT scan of the chest, abdomen and pelvis with intravenous and oral contrast. Patients also underwent a mandatory upper endoscopy with endoscopic ultrasound and bone scan. Most patients were also evaluated with either a positron emission tomography (PET) scan (21 patients) or single-photon emission computed tomography (SPECT) scan (7 patients).

Prior chemotherapy, surgery, or radiation therapy for esophageal cancer was not allowed. Other eligibility criteria included an adequate performance status (Eastern Cooperative Oncology Group Performance Status ≤1), a serum creatinine ≤ 1.5 mg/dL, serum bilirubin ≤ 1.5 mg/dL, and aspartamine transaminase ≤ 3 times the upper limit of normal. Patients were ineligible if they had a history of prior severe reaction to nonsteroidal anti-inflammatory drugs (NSAIDs) or sulfonamides or had significant comorbidities that made chemoradiation inadvisable. Patients with another active malignancy, Gilbert’s Disease, interstitial pulmonary fibrosis, seizure disorder requiring anti-epileptics, uncontrolled diarrhea, symptomatic hearing loss, and grade 2–4 neuropathy were also excluded.

This trial was approved by the Internal Review Board (IRB) of the Dana-Farber/Harvard Cancer Center. All patients signed an IRB-approved consent prior to enrollment.

### Treatment plan

All patients were scheduled to receive neoadjuvant chemoradiation, perioperative celecoxib, and surgery. Neoadjuvant therapy consisted of external beam radiation (5040 cGy) delivered in 28 fractions over 5.5 weeks along with intravenous cisplatin 30 mg/m^2^ and irinotecan 65 mg/m^2^ on weeks 1, 2, 4, and 5. Patients started celecoxib 400 mg by mouth twice daily 3 days prior to initiation of chemoradiation and stopped 1 week before surgery. Surgery was performed 4 to 8 weeks following the completion of chemoradiation. An esophagectomy was performed according to normal standard of care practices at Brigham and Women’s Hospital or at Massachusetts General Hospital. Celecoxib was restarted at the same dose and schedule upon discharge from the hospital following esophagectomy. Patient then continued celecoxib for 26 weeks.

### Study design and assessments

Toxicity assessments were made according to the National Cancer Institute’s common toxicity scale (Version 1.0) and the RTOG Radiation Morbidity Scoring Criteria.

Cisplatin was dose reduced by 50 % if the serum creatinine was between 1.7 and 2.0 mg/dL. Cisplatin was temporarily stopped and subsequently dose reduced by 50 % for a serum creatinine > 2.0 mg/dL, grade 3–4 ototoxicity, and grade 3–4 neuropathy. Irinotecan was held and subsequently dose reduced to 50 mg/m^2^ for an absolute neutrophil count (ANC) <1000/mm^3^, platelets <75,000/mm^3^, grade 3–4 diarrhea, and grade 4 fatigue. If treatment parameters were not met for either cisplatin or irinotecan, treatment with both chemotherapy drugs was interrupted. Radiation was temporarily stopped for ANC <1000/mm^3^, platelets <50,000/mm^3^, any grade 4 toxicity, and grade 3 esophagitis/mucositis. Radiation was also postponed in instances where irinotecan/cisplatin was held for ≥ 2 weeks. Celecoxib was held for any grade 4 toxicity, grade 3 gastric or duodenal ulcers, vomiting, or bleeding.

### Statistical analyses

The primary objective of this trial was to determine the pathological complete response rate and the toxicities of the chemoradiation regimen. A pathological complete response was defined as the absence of tumor cells in the esophagectomy specimen. Our analysis defined tumor downstaging as a decrease in the stage seen on pathological staging following esophagectomy compared to the stage determined during pretreatment staging workup.

The trial had a two-stage design. In the first stage, if at least 3 of 17 patients had a pathological complete response, the second stage of patients was allowed to enroll. A pathological complete response rate of 25 % was chosen as a benchmark that would be promising in this population because, at the time of the study design, this was the approximate average pathological complete response rate seen on combined modality trials. The study population of 40 patients was selected because the probability that the combination would be considered promising was 80 % if the true complete pathological response rate was 25 %. In addition, a study population of 40 patients was selected because the 90 % confidence interval on any toxicity rate would be no wider than 30 percentage points and the chance of observing one or more rare (5 % incidence) toxicities was greater than 83 %.

Secondary objectives of the protocol included measuring the median overall survival and median progression free survival. Progression free survival and overall survival were determined by the Kaplan-Meier method. Both progression free survival and overall survival were analyzed with an intention to treat analysis. Overall survival was calculated as the time from enrollment until death, and progression free survival as the time until disease progression or death. Survival calculations based on pathological staging, measured the amount time from surgery until progression of disease or death.

## Results

### Baseline patient characteristics

From January 2002 until September 2005, 40 patients with locally advanced esophageal or gastroesophageal junction cancer were enrolled. Baseline characteristics of patients enrolled in the study are shown in Table 1. The median age was 65 years and the majority of patients were male (85 %). Eighty-five percent of the patients had adenocarcinoma pathology. Most of the tumors were located in either the distal esophagus (57.5 %) or gastroesophageal junction (25 %). Preoperative staging showed that 30 % of tumors were stage IIA, 20 % stage IIB, 22.5 % stage III, and 27.5 % stage IVA (AJCC, 5th Edition).

**Table 1:** Baseline Patient Characteristics

Characteristic	Total (*N* = 40)
Age	
Median	65 years
Range	37 to77 years
Gender	
Male	34 (85 %)
Female	6 (15 %)
Performance Status	
ECOG 0	19 patients (48 %)
ECOG 1	21 patients (52 %)
Pathology	
Adenocarcinoma	34 patients (85 %)
Squamous carcinoma	6 patients (15 %)
Stage (AJCC 5th edition)	
Stage IIA	12 patients (30 %)
Stage IIB	8 patients (20 %)
Stage III	9 patients (22.5 %)
Stage IVA	11 patients (27.5 %)
Tumor Location	
Middle Esophagus	7 patients (17.5 %)
Lower Esophagus	23 patients (57.5 %)
Gastroesophageal Junction	10 patients (25 %)
Dysphagia (Grade ≥1)	34 patients (85 %)
Greater than 10 % weight loss	10 patients (25 %)

### Treatment duration and toxicities

All 40 patients initiated treatment with neoadjuvant chemoradiation and celecoxib. Toxicity assessment and efficacy analyses were performed on all 40 patients. There were no treatment-related deaths during chemoradiation. Four patients (10 %) discontinued protocol therapy after 2 to 3 weeks due to treatment-related toxicity. One of these patients developed febrile neutropenia and sepsis. The other three patients had severe dehydration secondary to severe nausea and/or diarrhea. In addition, one patient could not complete his chemoradiation following the development of a paraneoplastic neurologic syndrome associated with anti-Yo antibodies and was taken to surgery early. During chemoradiation, four patients (10 %) required a radiation delay and five patients (12.5 %) required chemotherapy dose reduction, primarily for neutropenia and diarrhea.

Grade 3 and 4 toxicities observed during chemoradiation are listed in Table 2. The most common treatment-related grade 3–4 toxicities were dysphagia (20 %), anorexia (17.5 %), dehydration (17.5 %), nausea (15 %), neutropenia (12.5 %), diarrhea (10 %), and fatigue (7.5 %). Three patients (7.5 %) developed febrile neutropenia.

**Table 2:** Treatment-Related Preoperative Grade 3 or 4 Adverse Events

Adverse Event	Grade 3	Grade 4
Hematologic		
Leukopenia	6 (15.0 %)	2 (5.0 %)
Neutropenia	3 (7.5 %)	2 (5.0 %)
Anemia requiring blood transfusion	2 (5.0 %)	
Febrile neutropenia	1 (2.5 %)	2 (5.0 %)
Thrombocytopenia		1 (2.5 %)
Gastrointestinal		
Dysphagia, esophagitis, odynophagia	7 (17.5 %)	1 (2.5 %)
Anorexia	7 (17.5 %)	
Dehydration	7 (17.5 %)	
Nausea	6 (15.0 %)	
Diarrhea	4 (10.0 %)	
Vomiting	4 (10.0 %)	
Abdominal pain or cramping	2 (5.0 %)	
Systemic		
Fatigue	3 (7.5 %)	
Hypotension	3 (7.5 %)	1 (2.5 %)
Hyperbilirubinemia	1 (2.5 %)	
Creatinine Elevation	1 (2.5 %)	
Chest pain (non-cardiac)	1 (2.5 %)	
Supraventricular arrhythmia	1 (2.5 %)	
Syncope	1 (2.5 %)	
Thrombosis/embolism		1 (2.5 %)

### Surgery

After completion of chemoradiation, one patient did not undergo surgery because of progressive disease and a second patient was deemed surgically unresectable. Of the 38 patients who proceeded to esophagectomy, 23 patients (60.5 %) received a 3-hole esophagectomy, 7 patients completed an Ivor Lewis esophagectomy (18.4 %), 5 patients underwent a transhiatal esophagectomy (13.1 %), and 3 patients had a thoracoabdominal esophagectomy (7.8 %).

Following surgery, patients were hospitalized for a median of 11 days (range 7 to 62 days). Eight patients (21 %) were hospitalized for more than 14 days. One patient developed an anastomotic leak and died from respiratory failure 59 days after esophagectomy. Two patients died of sepsis 61 days and 137 days after esophagectomy. In addition to these three deaths, two patients developed a chylothorax that required surgical repair.

### Pathologic response and survival assessment

Thirteen out of the 40 patients (32.5 %) had a pathologic complete response (Table 3). Another 15 % of patients had microscopic residual disease. Tumor downstaging occurred in 65 % of the 40 patients enrolled in the study. Thirty-five of the thirty eight patients (92.1 %) who underwent esophagectomy had an R0 resection.

**Table 3:** Pathological Staging after Surgery

Stage	Total 40 Patients
Complete Response	13 patients (32.5 %)
Microscopic Residual Disease	6 patients (15 %)
Stage I	3 patients (7.5 %)
Stage IIA	4 patients (10 %)
Stage IIB	4 patients (10 %)
Stage III	5 patients (12.5 %)
Stage IVA	3 patients (7.5 %)
Stage IVB or Refused Surgery	2 patients (5 %)

**Fig. 1:**
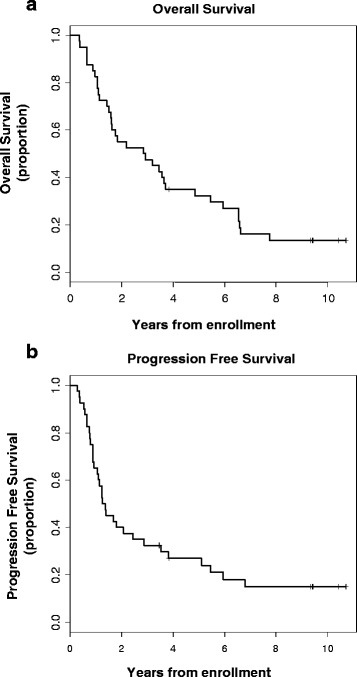
Kaplan-Meier estimates of overall survival (**a**) and progression free survival (**b**) for all 40 patients enrolled in the trial

All 40 patients were assessed according to an intention to treat analysis. One patient was lost to follow-up at 3.9 years after enrollment. Patients enrolled in the trial had a median progression free survival of 15.7 months (95 % confidence interval (CI), 11.0 to 29.3 months) and a median survival of 34.7 months (95 % CI, 19.0 to 44.5 months) (Fig. 1). The three-year survival rate was 47.5 % (95 % CI, 31.6–61.8 %) and the five-year survival rate was 30 % (95 % CI, 18.6–46.8 %). Three deceased patients had no evidence of esophageal cancer recurrence and died of causes other than esophageal cancer (stroke, pneumonia, and colon cancer).

**Fig. 2:**
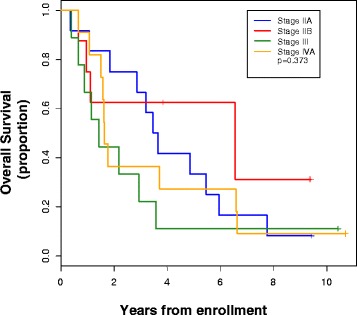
Kaplan-Meier estimates of overall survival for all 40 patients by pretreatment (clinical) stage (AJCC, 5th Edition)

Figure 2 shows the Kaplan-Meier curves of clinical pretreatment stage specific survival rates. Preoperative clinical stage was not predictive of overall survival (P, log-rank =0.373). Median overall survival for stage IIA was 3.6 years (95 % CI, 1.1 to 5.9 years), stage IIB 6.5 years (95 % CI, 0.7 years to non-estimable), stage III 1.42 years (95 % CI, 0.4 to 3.6 years), and stage IVA 1.6 years (95 % CI, 1.07 to 6.6 years). There was no significant difference in the overall survival rate of patients with adenocarcinoma and patients with squamous cell carcinoma (data not shown).

**Fig. 3:**
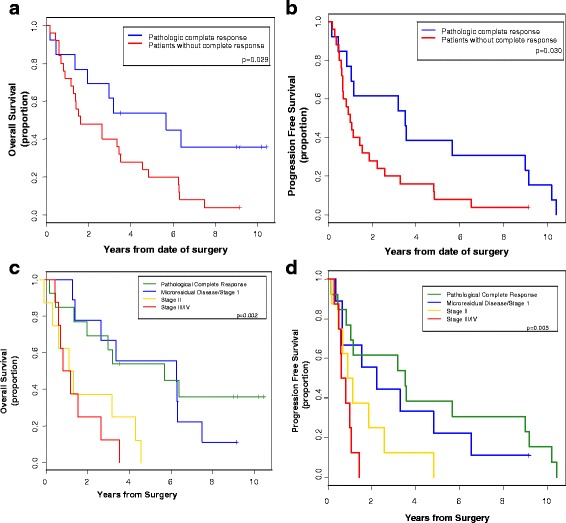
Kaplan-Meier estimates of overall survival (**a**) and progression free survival (**b**) in patients with and without a complete pathologic complete response. **c** and **d** are Kaplan-Meier estimates of overall surival (**c**) and progression free survival (**d**) of four groups based on pathologic staging (AJCC, 5th Edition). The four groups are patients with a pathologic complete response, microscopic residual disease or stage I, stage II, or stage III/IV cancers by pathologic staging

When compared to patients without a pathological complete response, those with a pathological complete response had a statistically significant increase in median survival (5.7 vs. 1.6 years, respectively, P, log-rank = 0.029) and progression-free survival (3.5 vs. 1.0 years, respectively, P, log-rank = 0.030) (Fig. 3a and b). In an exploratory analysis, we used pathological staging defined after surgical resection to segregate patients into four groups: patients with (1) pathological complete response, (2) microscopic residual disease or stage I, (3) stage II, or (4) stage III/IV cancers. There was a statistically significant difference in overall survival (*p* = 0.005) and progression free survival (*p* = 0.005) between each of these four groups (Fig. 3c and d).

Of the 26 patients with recurrent disease, at the time of initial detection of recurrence, 20 patients had distant recurrence (77 %), six patients had local recurrence (23 %), and one patient had simultaneous distant and local recurrence. Three of the six patients with local recurrence had received attenuated chemoradiation and one patient with local recurrence had refused surgery. Two of the 26 recurrences occurred five or more years after enrollment. One patient developed metastatic supraclavicular and mediastinal lymphadenopathy 5.1 years after enrollment and the other patient developed a metastatic lung lesion 6.8 years after enrollment. Both recurrences were biopsied and confirmed to be recurrent esophageal cancer.

At the time of final analysis, five of the 40 patients (12.5 %) were still alive at 9.6 years to 11 years since enrollment. Four of these five survivors had a complete response to neoadjuvant chemoradiation.

### Incidence of brain metastases

Brain metastases developed in 6 of the 40 patients on this trial (15 %) and were the first site of recurrence in four of these patients. Consistent with the standard of care, baseline brain imaging and routine CNS surveillance was not utilized on this trial. However, one patient’s cerebellar metastasis was detected on a surveillance PET/CT scan. All of the other patients’ brain metastases were discovered because the patients presented with symptoms suspicious for brain metastases.

Brain metastases occurred a median of 13.9 months after surgery (range, 8.0 to 31.1 months). Brain metastases were seen in 5 of 34 patients (15 %) with adenocarcinoma and 1 of 6 patients (17 %) with squamous cell carcinoma. The patient with squamous cell carcinoma had a tumor that originated in the middle of the esophagus, while all of the adenocarcinoma patients had tumors that originated in the distal esophagus. Three of the six patients (50 %) with brain metastases had an excellent response to neoadjuvant chemoradiation - with either a complete pathological response (two patients) or microscopic residual disease (one patient). Traditional risk factors for recurrence, including clinical stage, operative stage, or differentiation did not appear to predict metastases to the brain.

## Discussion

In this phase II trial, patients with locally advanced surgically resectable esophageal cancer received neoadjuvant chemoradiation using cisplatin/irinotecan in conjuction with celecoxib. The addition of celecoxib to the neoadjuvant cisplatin/irinotecan chemoradiation appeared to be well tolerated. The toxicity profile for patients treated with the combination of celecoxib with neoadjuvant irinotecan/cisplatin chemoradiation was similar to that seen in prior trials of neoadjuvant chemoradiation with irinotecan and cisplatin [13, 14].

Two previous studies examined neoadjuvant chemoradiation with cisplatin and irinotecan in patients with locally advanced, resectable esophageal cancer and reported a pathologic complete response rate of 16 % [13, 14]. While the addition of celecoxib to this regimen in our trial found a higher pathologic complete response rate (32.5 %), the median survival in our patients treated with concurrent celecoxib (34.7 months) was comparable to the results for neoadjuvant chemoradiation with cisplatin and irinotecan alone (31.7 and 36 months) [13, 14]. When our trial was initially designed, we felt a pathologic complete response rate of 25 % would be a promising result. However, pathologic complete response rate is a surrogate end-point and overall survival is clearly the most important measure of success. The survival rate in our trial is comparable to that of historical controls. These results would suggest that addition of celecoxib to this chemoradiation regimen is unlikely to meaningfully improve the efficacy of neoadjuvant chemoradiation with cisplatin/irinotecan.

There have been several other trials that have examined the addition of celecoxib to neoadjuvant treatment in esophageal cancer (Table 4) [31–33]. Similar to our results, all of these trials found that the addition of celecoxib to neoadjuvant therapy was well tolerated. However, Altorki et al. noted that the rate of venous thromboembolic events was higher than expected in the perioperative period [31].

**Table 4:** Trials Testing Preoperative Celecoxib in Esophageal Cancer

Study	Phase	Regimen	Patients	Conclusion
Dawson et al. [33]	1	5-FU/Cisplatin Chemoradiation Combined with Celecoxib	13	Regimen was well tolerated. The study was closed early because of external safety concerns regarding Celecoxib.
Altorki et al. [31]	2	Carboplatin/Paclitaxel Chemotherapy Combined with Celecoxib	39	Regimen was well tolerated with the exception of the fact that the rate perioperative venous thromboembolic events was higher than expected. Patients with tumors that expressed COX2 demonstrated higher rates of major pathological response and improved overall survival.
Govindan et al. [32]	2	5-FU/Cisplatin Chemoradiation Combined with Celecoxib	31	Regimen was well tolerated. The pathological complete response rate of 5-FU/Cisplatin/Celecoxib chemoradiation was similar to historical controls.

Consistent with our results, the only phase 2 esophageal cancer study examining celecoxib in combination with neoadjuvant chemoradiation concluded that the addition of celecoxib did not increase the efficacy of the chemoradiation [32]. Another study, examining neoadjuvant carboplatin/paclitaxel chemotherapy combined with celecoxib, met its primary endpoint by achieving complete pathological response and/or minimal residual disease in 12.8 % of its patients [31]. In an unplanned post-hoc analyses, this study found that COX2 expressing tumors had higher rates of major pathological response and improved overall survival [31].

Increased COX2 expression in esophageal cancer has been associated decreased survival [20–22, 34]. Preclinical models have demonstrated that COX2 may play a functional role in the malignant transformation from Barrett’s esophagus to esophageal adenocarcinoma [23, 24, 35, 36]. Multiple reports have suggested that cyclooxygenase inhibitors (COX inhibitors) decrease the risk of esophageal cancer [37–39]. One recent meta-analysis showed that COX inhibitors reduce the risk of developing esophageal adenocarcinoma by 30 % [40]. In addition, several preclinical studies have shown that the selective COX2 inhibitor celecoxib works synergistically with radiation to increase cancer cell death [25, 26, 41]. Multiple reports have demonstrated that high expression of COX2 correlates with decreased responsiveness to radiation [27–29, 42]. Nonetheless, despite these encouraging preclinical data and observational studies in humans, we do not find a clear survival benefit when comparing our regimen combining celecoxib with cisplatin/irinotecan chemoradiation to prior trials of cisplatin/irinotecan chemoradiation alone.

A major strength of our trial is the availability of long-term follow-up data. The three- and five-year survival rates, 47.5 and 30 % respectively, seen in our study appear comparable to other studies [43, 44]. Highlighting the importance of neoadjuvant chemoradiation, the five long-term surviving patients in our study all had an excellent response to chemoradiation. Four of the five patients (80 %) had a complete response to chemoradiation and the other patient’s tumor was downsized to T1N0. In fact, pathologic staging at the time of surgery was a significant predictor of both progression free survival and overall survival (Fig. 3c and d).

A weakness of this paper is that it utilizes cisplatin/irinotecan chemoradiation. While neoadjuvant cisplatin/irinotecan chemoradiation was a commonly used regimen at the time this study was conducted, since the publication of the CROSS trial, the standard of care has been carboplatin and paclitaxel [10]. Another limitation of this trial is that it is a single arm 40 patient study. An additional weakness of this study is that there is no data about celecoxib compliance.

Despite the notion that the brain represents an uncommon site of metastasis in esophageal cancer, 15 % of patients enrolled in our trial developed brain metastases during follow-up. While a series of 1588 esophageal cancer patients found only a 1.7 % prevalence of brain metastases [45], more recent series that examined the incidence of brain metastases following neoadjuvant or adjuvant treatment for esophageal cancer reported rates of 13 to 18 % [46, 47]. Of note, in the current trial, response to neoadjuvant therapy did not predict the development of brain metastases.

## Conclusion

In conclusion, the addition of celecoxib did not appear meaningfully improve the efficacy of neoadjuvant chemoradiation in an unselected population of locally advanced esophageal cancer patients. Given the high rate of recurrence and poor outcome in this patient population, future studies need to define more effective neoadjuvant and adjuvant regimens.

## Abbreviations

ANC, Absolute neutrophil count; AJCC, American Joint Committee on Cancer; COX inhibitors, Cyclooxygenase inhibitors; COX2, Cyclooxygenase 2; IRB, Internal Review Board; NSAID, Non-steroidal anti-inflammatory; SPECT, Single-photon emission computed tomography
